# P-2027. Advancing Outpatient Infectious Disease Care for Oncology Patients; A Study on Continuity of Care and Clinic Expansion

**DOI:** 10.1093/ofid/ofaf695.2191

**Published:** 2026-01-11

**Authors:** Joshua M Modrick, Ryan Nazareno, Sikander Chohan, Lea M Monday

**Affiliations:** Wayne State University/Detroit Medical Center, Detroit, MI; Wayne State University School of Medicine, Detroit, Michigan; Wayne State University/Detroit Medical Center, Detroit, MI; Wayne state University School of Medicine, Detroit, Michigan

## Abstract

**Background:**

Infectious complications are common during cancer treatment and lead to delays in optimal care. At our center, an NCI-designated comprehensive cancer institute, outpatient infectious disease (ID) care for oncology patients traditionally depended on the malignancy type. Hematologic malignancies were managed at the cancer center, while solid organ tumor patients were seen at off-site fellows’ clinic that also covered HIV.

This approach presented issues with geographic and EMR separation resulting in poor continuity of care. To address this, we performed a quasi-experimental quality improvement (QI) project to expand our cancer center-based clinic to include all cancer patients, regardless of malignancy type.

**Figure 1:**
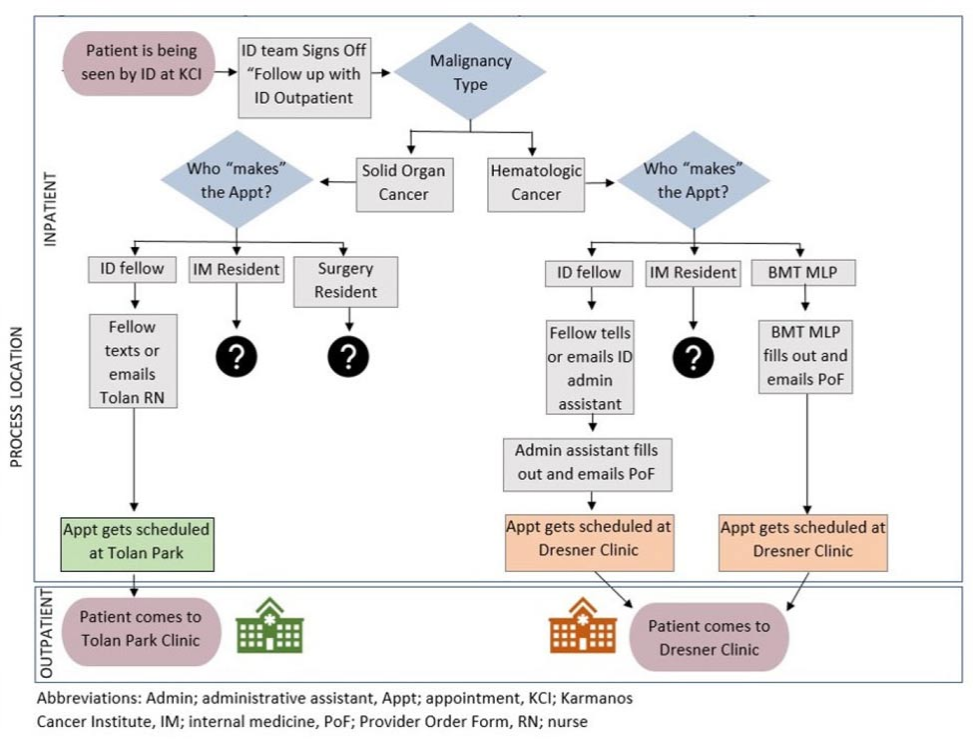
Process Map of Infectious Disease Outpatient Clinic Scheduling Process

**Figure 2:**
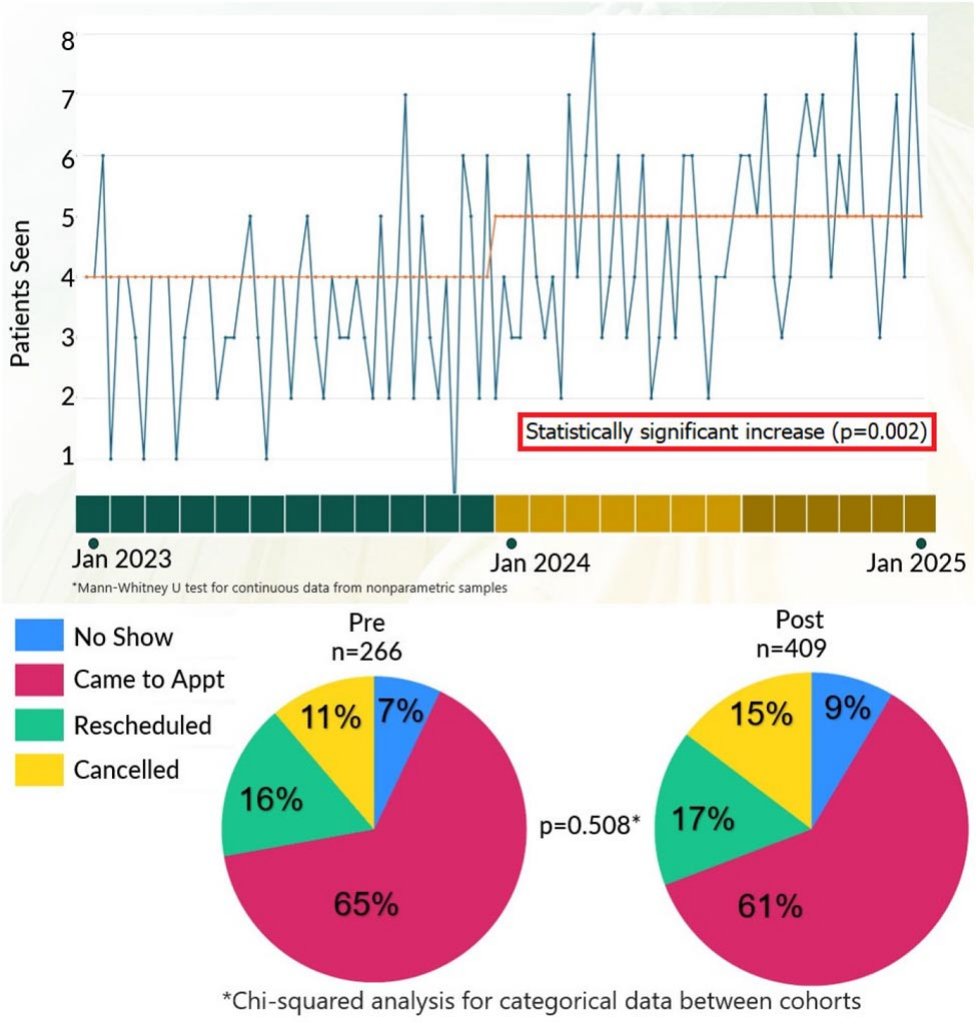
Run Chart of Patients Seen Per Week and Clinic Attendance

**Methods:**

From Jan 2023 to Dec 2023, our scheduling process was analyzed with QI tools including process map (Fig 1) and Ishikawa diagram. We engaged stakeholders and designated ID fellows as owners of a homogenized scheduling request process starting Jan 2024. After demonstrating need, clinical slots were expanded from 4 to 8 per session starting in August 2024. We then analyzed how our changes affected clinic volumes, show rates, and revenue (Fig 2).

**Figure 3:**
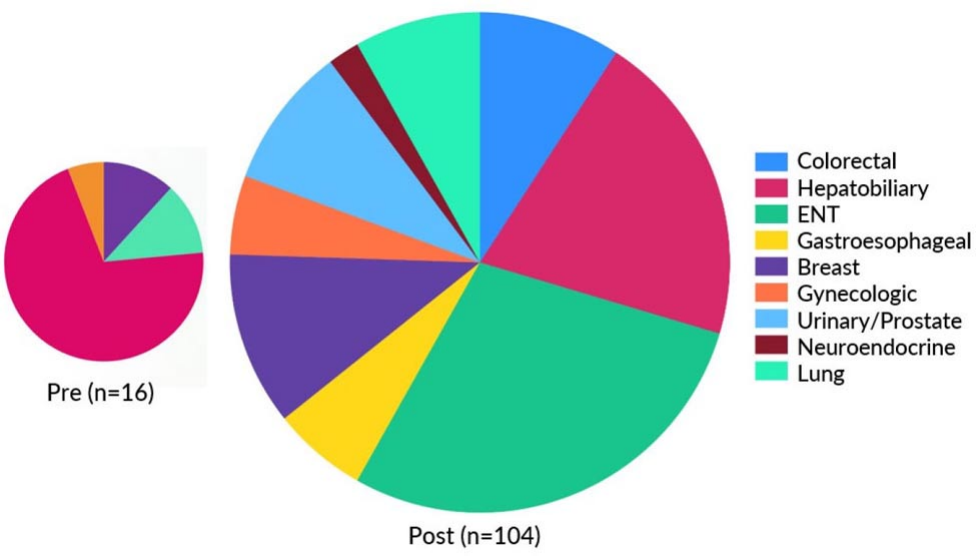
Types of Solid Organ Malignancies Supported

**Figure 4:**
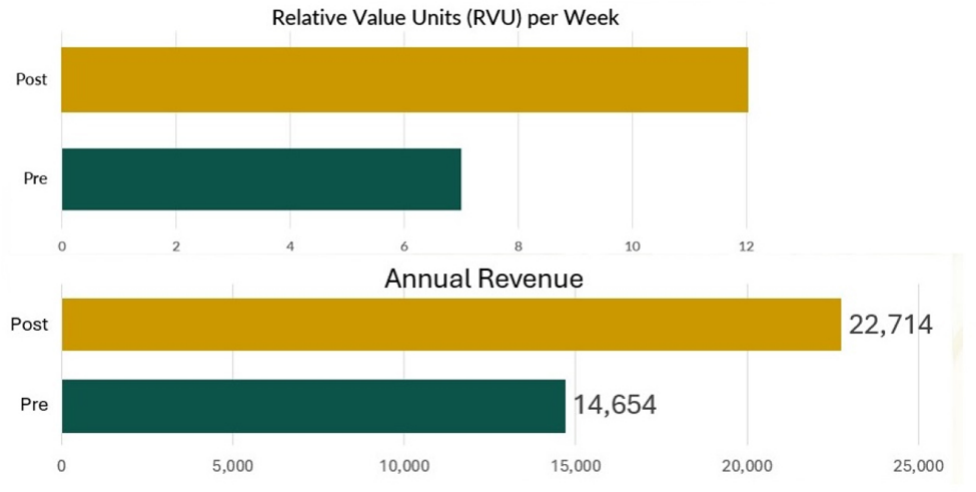
Financial Impact of Scheduling Changes

**Results:**

From Jan 2023 to Dec 2023, our scheduling process was analyzed with QI tools including process map (Fig 1) and Ishikawa diagram. We engaged stakeholders and designated ID fellows as owners of a homogenized scheduling request process starting Jan 2024. After demonstrating need, clinical slots were expanded from 4 to 8 per session starting in August 2024. We then analyzed how our changes affected clinic volumes, show rates, and revenue (Fig 2).

**Conclusion:**

Patients seen weekly in the clinic increased significantly (p=0.0002) from 266 to 409. No-show rates were not affected (p=0.508) and remained below 10% even after including solid tumor patients (Fig 2). ID clinic utilization was particularly robust for patients with head/neck and lung malignancies (Fig 3). Hematologic malignancy patient volumes were not negatively impacted. RVUs generated increased by 69%, with over $8,000 additional annual revenue (Fig 4).

**Disclosures:**

All Authors: No reported disclosures

